# Supplementary material for: Navigating from cellular phenotypic screen to clinical candidate: selective targeting of the NLRP3 inflammasome
Source: EMBO Mol Med. 2024 Dec 9;17(1):54–84. doi: 10.1038/s44321-024-00181-4 (PMC11730736; doi:10.1038/s44321-024-00181-4)
Supplement: Supplementary file 1 — Appendix [file 44321_2024_181_MOESM1_ESM.pdf]

# Appendix figures

## Table of content


| Appendix Figure | Page number | Title |
|---|---|---|
| Appendix Figure S1 | 2 | Schematical representation of the hit-finding plan |
| Appendix Figure S2 | 3 | Nano-DSF shows target engagement |
| Appendix Figure S3 | 4 | Data collection, reconstruction, and model refinement statistics of cryo-EM structures |
| Appendix Figure S4 | 5 | Cryo-EM analysis of NLRP3 with compound C. |
| Appendix Figure S5 | 6 | HDX and targeted amino-acid foot printing result of compound C binding to NLRP3 |
| Appendix Figure S6 | 7 | ATPase activity is dose-dependently reduced using different compounds |
| Appendix Figure S7 | 8 | ASC Speck in THP-1 cells |
| Appendix Figure S8 | 9 | Strategy for the generation of a conditional Nlrp3 mouse and functional validation of the Nlrp3 full body knock-out mice using BMDMs |
| Appendix Figure S9 | 10 | Strategy and validation for the generation of a tamoxifen-inducible CreERT2 mouse model |
| Appendix Figure S10 | 11 | Sequence and validation of THP-1 knock-in cells introducing A354V mutation in the *NLRP3* gene |

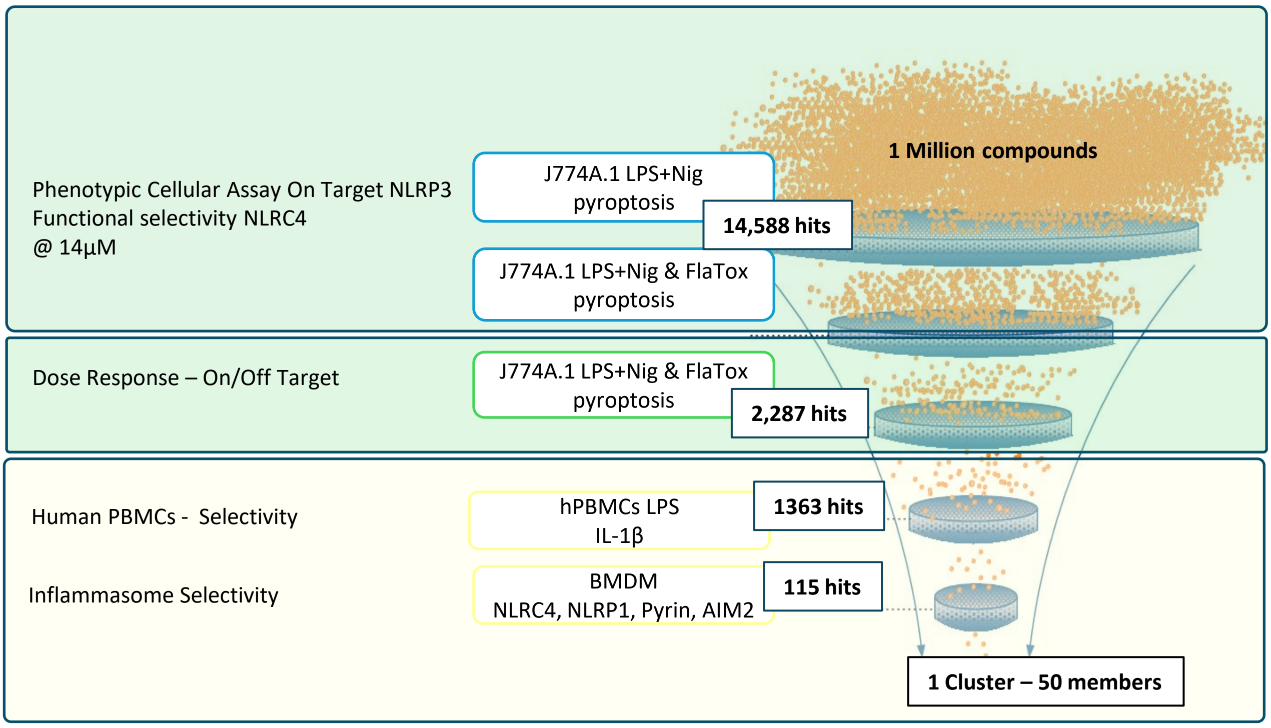


**Appendix Figure S1. Schematical representation of the hit-finding plan.**

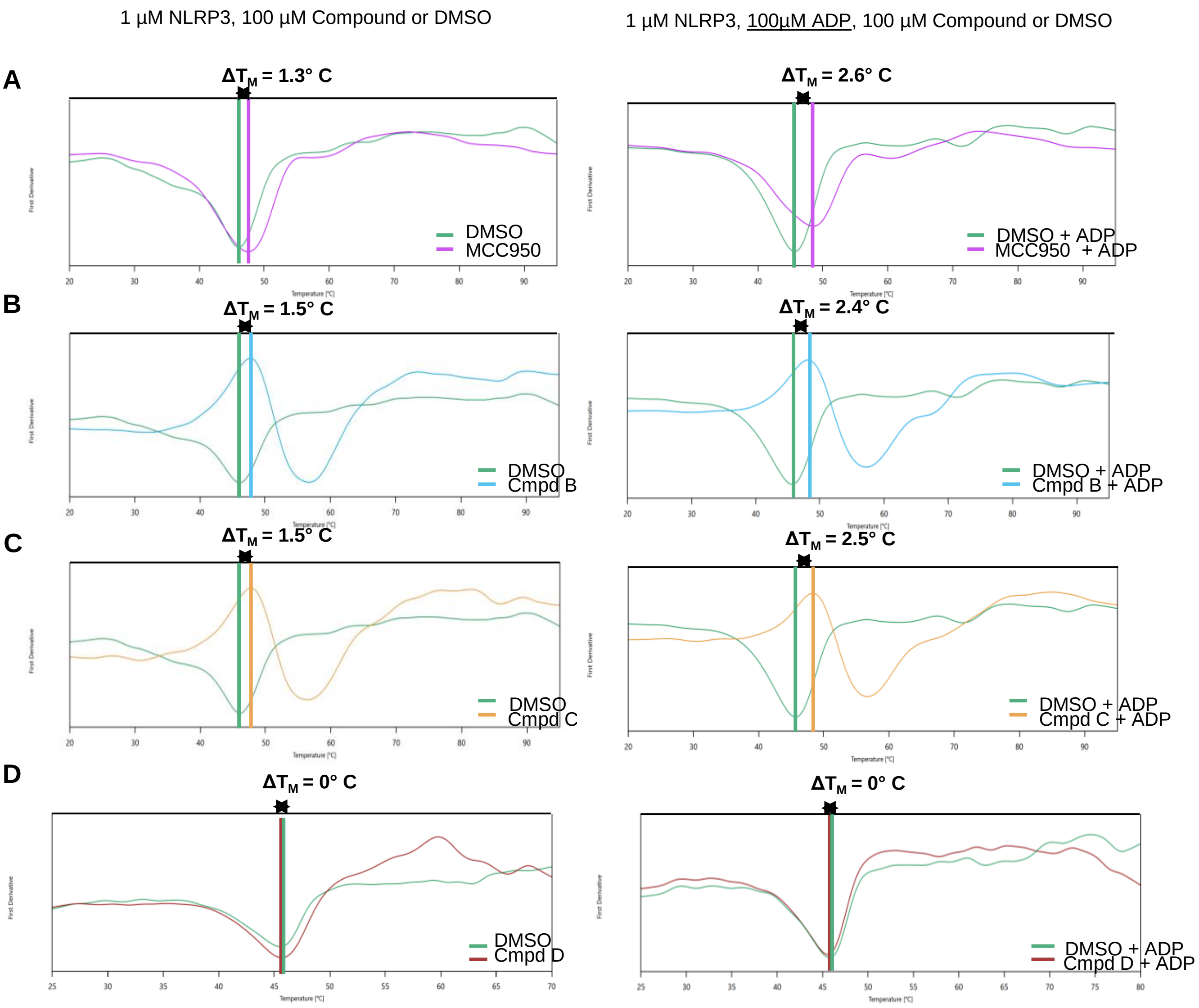


**Appendix Figure S2. Nano-DSF shows target engagement.** Reference compound MCC950 (A), compound B (B) and C (C) and D (D) show direct interaction to deltaPYD-hNLRP3 +/- ADP.



Appendix Figure S2

| | NLRP3 + cmpd C |
|---|---|
| **Data collection** | |
| **Microscope** | Titan Krios |
| **Voltage (keV)** | 300 |
| **Nominal magnification** | 130000 x |
| **Exposure navigation** | Image Shift |
| **Electron exposure (e /Å²)** | 44.57 |
| **Total exposure time (sec)** | 6 |
| **Detector** | K2 Summit |
| **Pixel size (Å)*** | 1.04 |
| **Defocus range (μm)** | -0.8 to -1.5 |
| **Micrographs Used** | 8,913 |
| **Final Refined particles (no.)** | 94,472 |
| | |
| **Reconstruction** | |
| **Symmetry imposed** | D2 |
| **Resolution (global)** | |
| **FSC 0.143** | 3.76 Å |
| **Applied B-factor (Å²)** | -159 |
| | |
| **Refinement** | |
| **Protein residues** | 4724 |
| **Ligand** | 12 |
| **Map Correlation Coefficient (Main chain)** | 0.8018 |
| **R.m.s deviations** | |
| **Bond lengths (Å)** | 0.0115 |
| **Bond angles (°)** | 1.05 |
| **Ramachandran** | |
| **Outliers** | 0.85 % |
| **Allowed** | 3.87 % |
| **Favored** | 95.28 % |
| **Rotamer outliers** | 0.00 % |
| **MolProbity score** | 1.30 |
| **EMRinger score** | 2.18 |
| **Clashscore (all atoms)** | 2.06 |

**Appendix Figure S3. Data collection, reconstruction, and model refinement statistics of cryo-EM structures**

*Calibrated pixel size at the detector

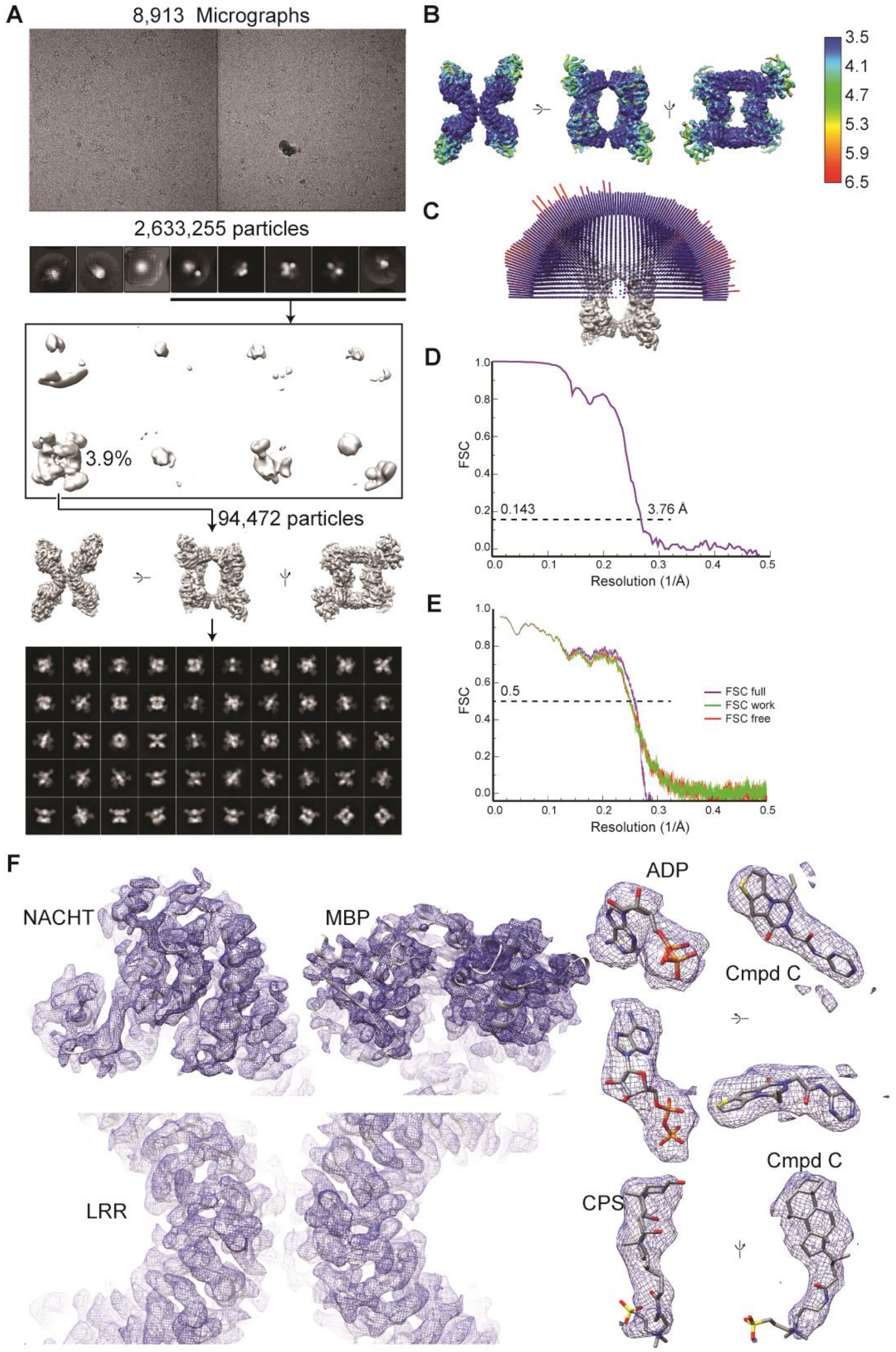


**Appendix Figure S4. Cryo-EM analysis of NLRP3 with compound C**. A) Flow chart of the cryo-EM data processing procedure. Details can be found in the Materials and methods. Representative cryo-EM micrographs were shown. B) Local resolution of the map estimated using the ResMap program and colored as indicated. C) Angular orientation distribution of the particles used in the final reconstruction. The particle distribution is indicated by different color shades. D) Fourier shell correlation (FSC) curve of the structure with FSC as a function of resolution using Relion output. The resolution is ~3.76 Å at the FSC cutoff of 0.143. E) Model validation. Comparison of the FSC curves between model and half map 1 (work), model and half map 2 (free), and model and full map are plotted in red, green and magenta, respectively. F) Sharpened cryo-EM density is displayed at the contour level 8σ for ADP, compound C, and CPS binding regions. The ADP, compound C, and CPS were shown as sticks.

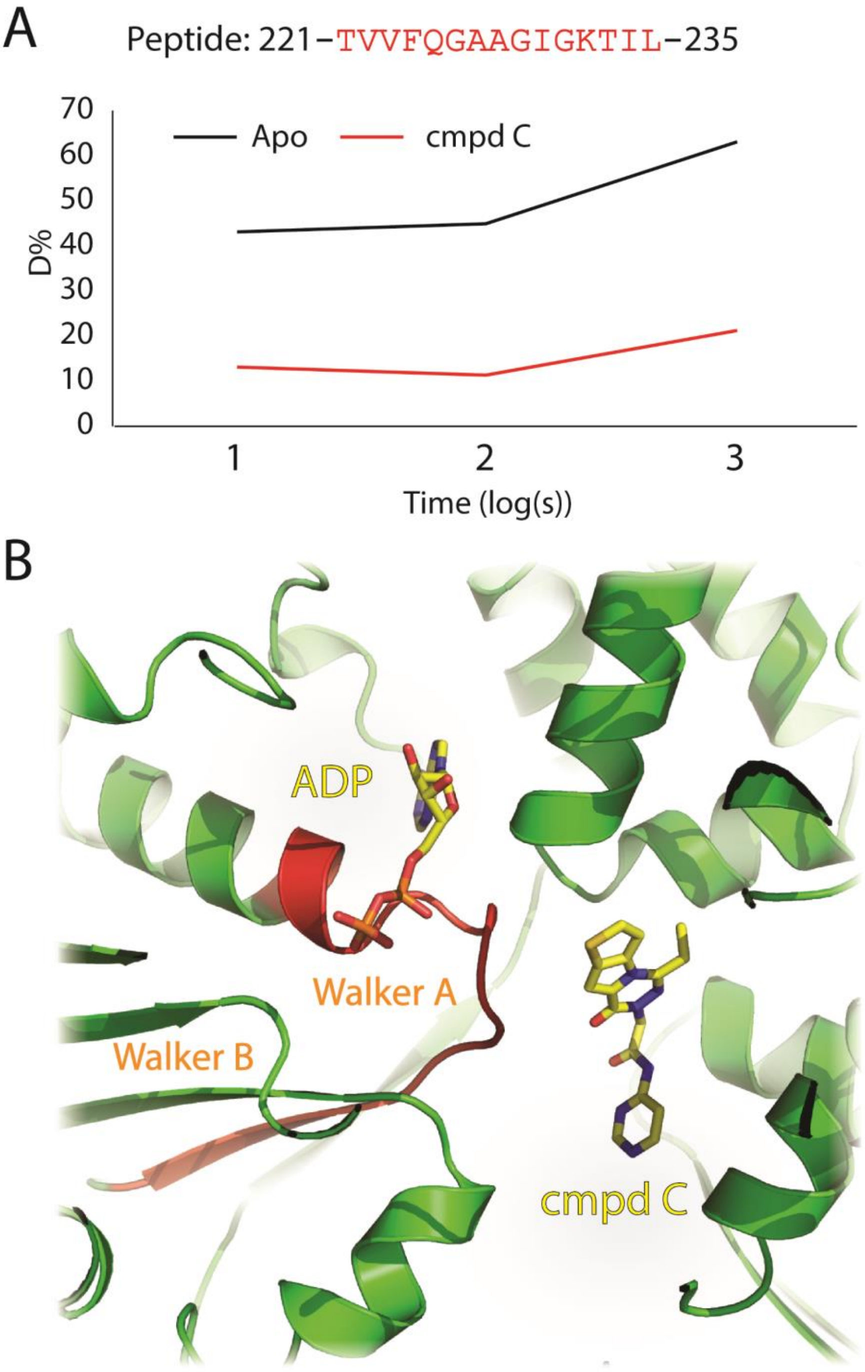


**Appendix Figure S5. HDX and targeted amino-acid foot printing result of compound C binding to NLRP3.** The protected regions upon compound C binding are mapped on the NACHT (cartoon representation) and colored in red (A). Representative HDX kinetic plots: black represents unbound state; red represents compound C bound state (B).

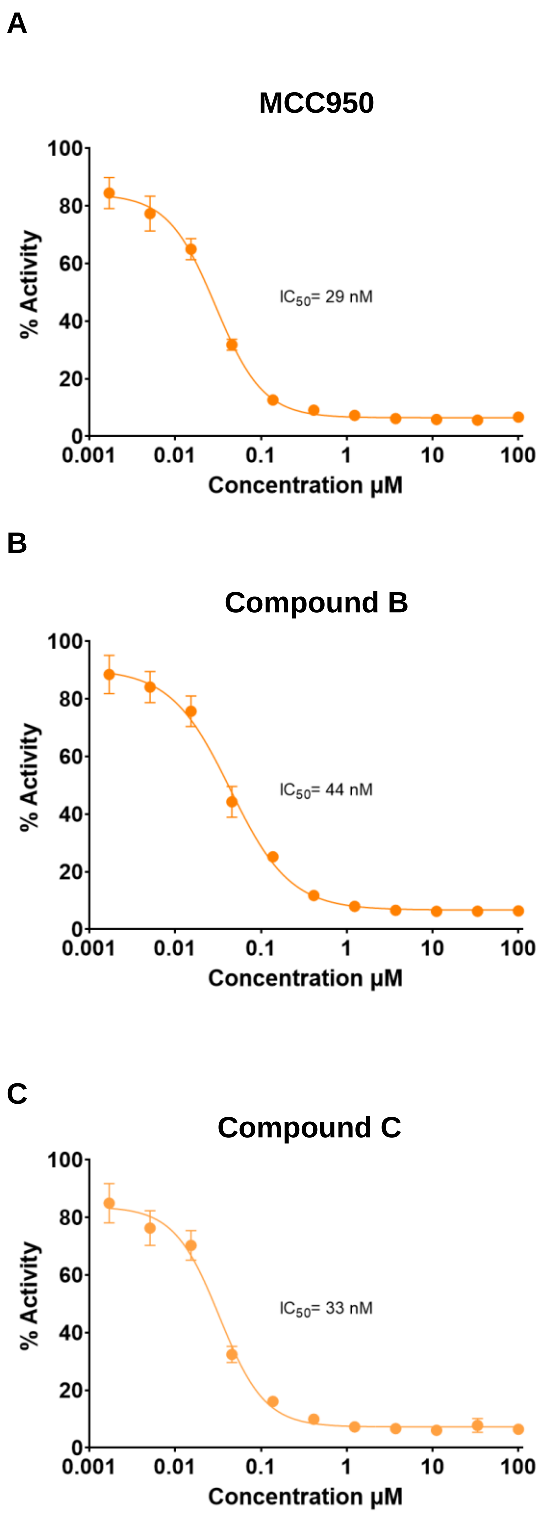


**Appendix Figure S6. ATPase activity is dose-dependently reduced using different compounds.** ATPase activity using reference compound MCC950 (A), compound B (B) and compound C (C) was determined.

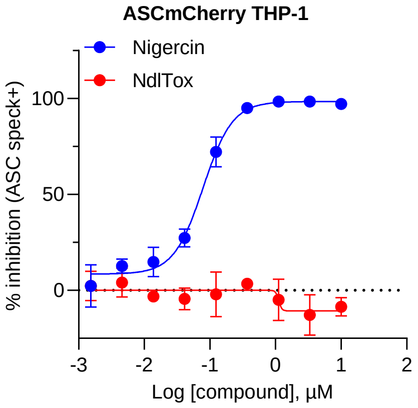


**Appendix Figure S7. ASC Speck in THP-1 cells.** ASC-mCherry expressing THP-1 cells were treated with 20 µM Nig or 200 ng/ml NdlTox and ASC specks formation was evaluated in the presence of compound A by incucyte. THP-1 cells were used (n=2) and representative image is shown depicting mean +/- SD of triplicate values.

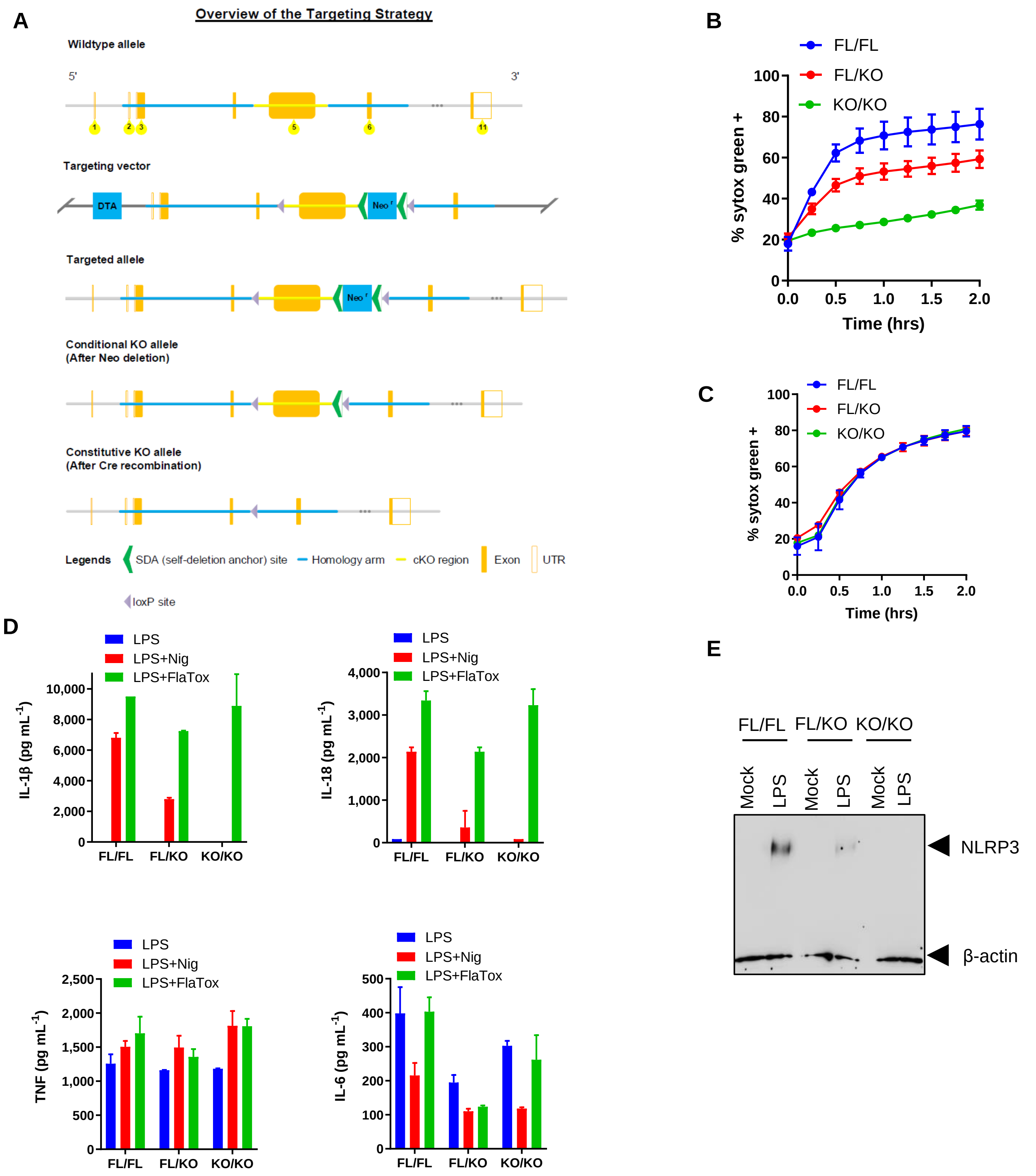


**Appendix Figure S8. Strategy for the generation of a conditional *Nlrp3* mouse and functional validation of the *Nlrp3* full body knock-out mice using BMDMs.** Vector design depicting the used strategy to generate the *Nlrp3* conditional knock-out mice (A), flox/flox, flox/KO and KO/KO BMDMs treated with LPS + nigericin (B) or LPS + FlaTox (C) and monitored for induction of cell death by Sytox green incorporation or cytokines released in the supernatant (D). Western blotting showing protein expression in untreated or LPS-treated BMDMs (E).

A

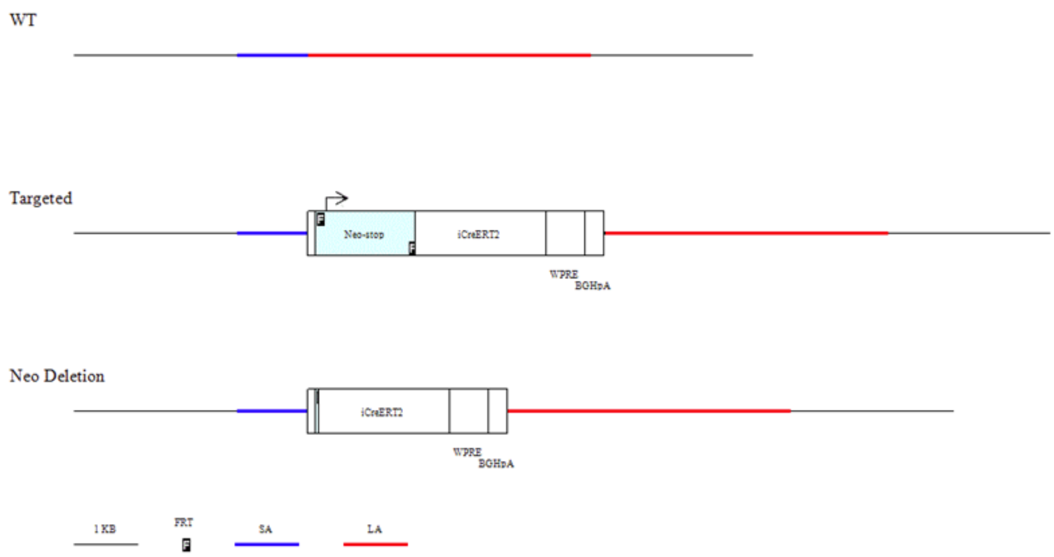


B

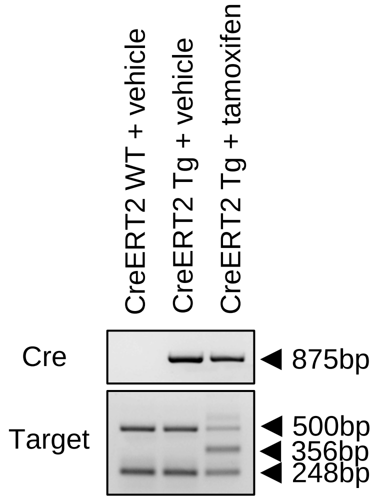


**Appendix Figure S9. Strategy and validation for the generation of a tamoxifen-inducible CreERT2 mouse model.** Vector design depicting the used strategy to generate targeted mice (A), genotyping results of animals crossed with the CreERT2 mice to generate a tamoxifen-inducible mutation (B).

**A** pLV[Exp]-Puro-TRE3G>{hNLRP3-6Ala-3xFLAG MWS A354V}

ATGAAGATGGCAAGCACCCGCTGCAAGCTGGCCAGGTACCTGGAGGACCTGGAGGATGTGGACTTGAAGAAA
TTTAAGATGCACTTAGAGGACTATCCTCCCCAGAAGGGCTGCATCCCCCTCCCGAGGGGTCAGACAGAGAAGG
CAGACCATGTGGATCTAGCCACGCTAATGATCGACTTCAATGGGGAGGAGAAGGCGTGGGCCATGGCCGTGT
GGATCTTCGCTGCGATCAACAGGAGAGACCTTTATGAGAAAGCAAAAAGAGATGAGCCGAAGTGGGGTTCAGA
TAATGCACGTGTTTCGAATCCCACTGTGATATGCCAGGAAGACAGCATTGAAGAGGAGTGGATGGGTTTACTG
GAGTACCTTTCGAGAATCTCTATTTGTAAAATGAAGAAAGATTACCGTAAGAAGTACAGAAAGTACGTGAGAAG
CAGATTCCAGTGCATTGAAGACAGGAATGCCCGTCTGGGTGAGAGTGTGAGCCTCAACAAACGCTACACACGA
CTGCGTCTCATCAAGGAGCACCGGAGCCAGCAGGAGAGGGAGCAGGAGCTTCTGGCCATCGGCAAGACCAA
GACGTGTGAGAGCCCCGTGAGTCCCATTAAGATGGAGTTGCTGTTTGACCCCGATGATGAGCATTCTGAGCCT
GTGCACACCGTGGTGTTCCAGGGGGCGGCAGGGATTGGGAAAACAATCCTGGCCAGGAAGATGATGTTGGAC
TGGGCGTCGGGGACACTCTACCAAGACAGGTTTGACTATCTGTTCTATATCCACTGTCGAGAGGTGAGCCTTG
TGACACAGAGGAGCCTGGGGGACCTGATCATGAGCTGCTGCCCCGACCCAAACCCACCCATCCACAAGATCG
TGAGAAAACCCTCCAGAATCCTCTTCCTCATGGACGGCTTCGATGAGCTGCAAGGTGCCTTTGACGAGCACAT
AGGACCGCTCTGCACTGACTGGCAGAAGGCCGAGCGGGGAGACATTCTCCTGAGCAGCCTCATCAGAAAGAA
GCTGCTTCCCGAGGCCTCTCTGCTCATCACCACGAGACCTGTGGTCCTGGAGAAACTGCAGCACTTGCTGGAC
CATCCTCGGCATGTGGAGATCCTGGGTTTCTCCGAGGCCAAAAGGAAAGAGTACTTCTTCAAGTACTTCTCTGA
TGAGGCCCAAGCCAGGGCAGCCTTCAGTCTGATTCAGGAGAACGAGGTCCTCTTCACCATGTGCTTCATCCCC
CTGGTCTGCTGGATCGTGTGCACTGGACTGAAACAGCAGATGGAGAGTGGCAAGAGCCTTGCCCAGACATCC
AAGACCACCACCGCGGTGTACGTCTTCTTCCTTTCCAGTTTGCTGCAGCCCCGGGGAGGGAGCCAGGAGCAC
GGCCTCTGCGCCCACCTCTGGGGGCTCTGCTCTTTGGCTGCAGATGGAATCTGGAACCAGAAAATCCTGTTTG
AGGAGTCCGACCTCAGGAATCATGGACTGCAGAAGGCGGATGTGTCTGCTTTCCTGAGGATGAACCTGTTCCA
AAAGGAAGTGGACTGCGAGAAGTTCTACAGCTTCATCCACATGACTTTCCAGGAGTTCTTTGCCGCCATGTACT
ACCTGCTGGAAGAGGAAAAGGAAGGAAGGACGAACGTTCCAGGGAGTCGTTTGAAGCTTCCCAGCCGAGACG
TGACAGTCCTTCTGGAAAACTATGGCAAATTCGAAAAGGGGTATTTGATTTTTGTTGTACGTTTCCTCTTTGGCC
TGGTAAACCAGGAGAGGACCTCCTACTTGGAGAAGAAATTAAGTTGCAAGATCTCTCAGCAAATCAGGCTGGA
GCTGCTGAAATGGATTGAAGTGAAAGCCAAAGCTAAAAAGCTGCAGATCCAGCCCAGCCAGCTGGAATTGTTC
TACTGTTTGTACGAGATGCAGGAGGAGGACTTCGTGCAAAGGGCCATGGACTATTTCCCCAAGATTGAGATCA
ATCTCTCCACCAGAATGGACCACATGGTTTCTTCCTTTTGCATTGAGAACTGTCATCGGGTGGAGTCACTGTCC
CTGGGGTTTCTCCATAACATGCCCAAGGAGGAAGAGGAGGAGGAAAAGGAAGGCCGACACCTTGATATGGTG
CAGTGTGTCCTCCCAAGCTCCTCTCATGCTGCCTGTTCTCATGGATTGGTGAACAGCCACCTCACTTCCAGTTT
TTGCCGGGGCCTCTTTTCAGTTCTGAGCACCAGCCAGAGTCTAACTGAATTGGACCTCAGTGACAATTCTCTGG
GGGACCCAGGGATGAGAGTGTTGTGTGAAACGCTCCAGCATCCTGGCTGTAACATTCGGAGATTGTGGTTGGG
GCGCTGTGGCCTCTCGCATGAGTGCTGCTTCGACATCTCCTTGGTCCTCAGCAGCAACCAGAAGCTGGTGGA
GCTGGACCTGAGTGACAACGCCCTCGGTGACTTCGGAATCAGACTTCTGTGTGTGGGACTGAAGCACCTGTTG
TGCAATCTGAAGAAGCTCTGGTTGGTCAGCTGCTGCCTCACATCAGCATGTTGTCAGGATCTTGCATCAGTATT
GAGCACCAGCCATTCCCTGACCAGACTCTATGTGGGGGAGAATGCCTTGGGAGACTCAGGAGTCGCAATTTTA
TGTGAAAAAGCCAAGAATCCACAGTGTAACCTGCAGAAACTGGGGTTGGTGAATTCTGGCCTTACGTCAGTCTG
TTGTTCAGCTTTGTCCTCGGTACTCAGCACTAATCAGAATCTCACGCACCTTTACCTGCGAGGCAACACTCTCG
GAGACAAGGGGATCAAACTACTCTGTGAGGGACTCTTGCACCCCGACTGCAAGCTTCAGGTGTTGGAATTAGA
CAACTGCAACCTCACGTCACACTGCTGCTGGGATCTTTCCACACTTCTGACCTCCAGCCAGAGCCTGCGAAAG
CTGAGCCTGGGCAACAATGACCTGGGCGACCTGGGGGTCATGATGTTCTGTGAAGTGCTGAAACAGCAGAGC
TGCCTCCTGCAGAACCTGGGGTTGTCTGAAATGTATTTCAATTATGAGACAAAAAGTGCGTTAGAAACACTTCAA
GAAGAAAAGCCTGAGCTGACCGTCGTCTTTGAGCCTTCTTGGGCAGCTGCAGCTGCAGCTGACTACAAAGACG
ATGACGACAAGGGAAGTGACTACAAAGACGATGACGACAAGGGAAGTGACTACAAAGACGATGACGACAAGTG
A

**B**

```
  1 ~~~~~~~~~~~~~AAAGAAGCTGCTTCCCGAGGCCTCTCTGCTCATCACCACGAGACCTGTGGTCCTGGAGAAACTGCAGCACTTGCTGGACCATCCTCG 87
                 ||||||||||||||||||||||||||||||||||||||||||||||||||#||||||||||||||||||||||||||||||||||||
301 GCAGCCTCATCANAAAGAAGCTGCTTCCCGAGGCCTCTCTGCTCATCACCACGAGACCTGTGGCCCTGGAGAAACTGCAGCACTTGCTGGACCATCCTCG 400

 88 GCATGTGGAGATCCTGGGTTTCTCCGAGGCCAAAAGGAAAGAGTACTTCTTCAAGTACTTCTCTGATGAGGCCCAAGCCAGGGCAGCCTTCAGTCTGATT 187
    ||||||||||||||||||||||||||||||||||||||||||||||||||||||||||||||||||||||||||||||||||||||||||||||||||||
401 GCATGTGGAGATCCTGGGTTTCTCCGAGGCCAAAAGGAAAGAGTACTTCTTCAAGTACTTCTCTGATGAGGCCCAAGCCAGGGCAGCCTTCAGTCTGATT 500

188 CAGGAGAACGAGGTCCTCTTCACCATGTGCTTCATCCCCCTGGTCTGCTGGATCGTGTGCACTG~~~~~~~~~~~~~~~~~~~~~~~~~~~~~~~ 251
    ||||||||||||||||||||||||||||||||||||||||||||||||||||||||||||||||
501 CAGGAGAACGAGGTCCTCTTCACCATGTGCTTCATCCCCCTGGTCTGCTGGATCGTGTGCACTGCCTNNNNNAAANNNNNNNNNNNNNNNNAAAA 595
```

**Appendix Figure S10. Sequence and validation of THP-1 knock-in cells introducing A354V mutation in the *NLRP3* gene.**

Vector sequence with mutation highlighted (A), Sanger sequencing result to confirm mutation (B).
