# Supplementary figures and images for: Navigating from cellular phenotypic screen to clinical candidate: selective targeting of the NLRP3 inflammasome
Source: EMBO Mol Med. 2024 Dec 9;17(1):54–84. doi: 10.1038/s44321-024-00181-4 (PMC11730736; doi:10.1038/s44321-024-00181-4)

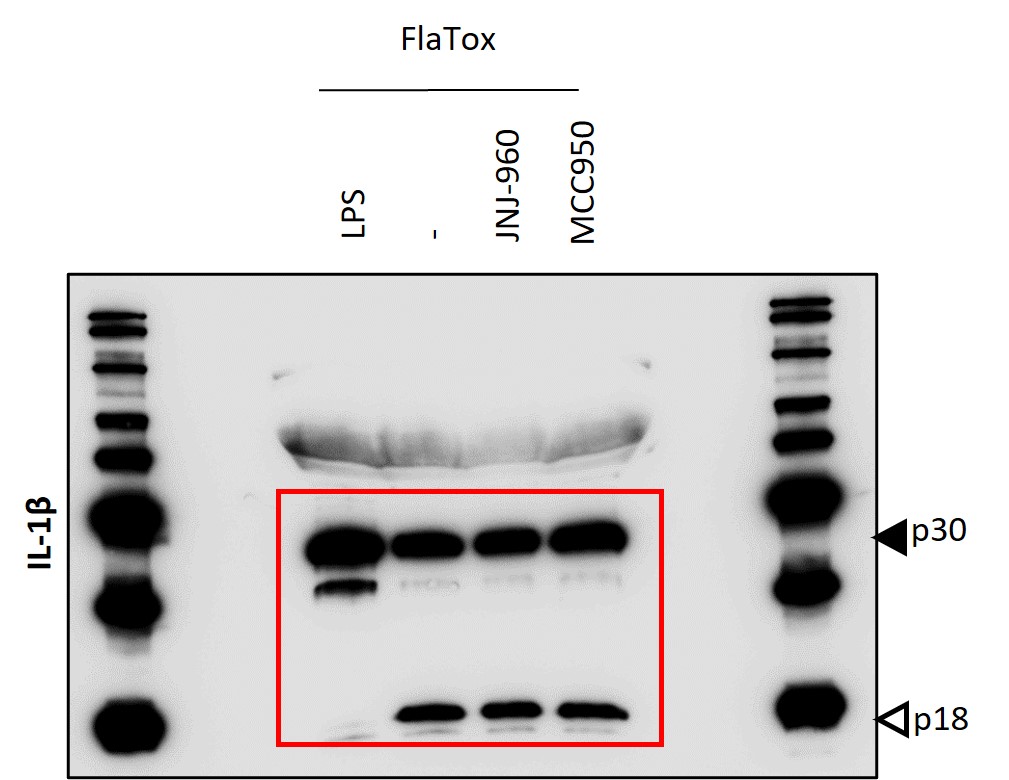

Supplement: Supplementary file 6 — Source data Fig. 4 [file 44321_2024_181_MOESM6_ESM.zip › Fig 4C_IL1b_FlaTox.jpg]

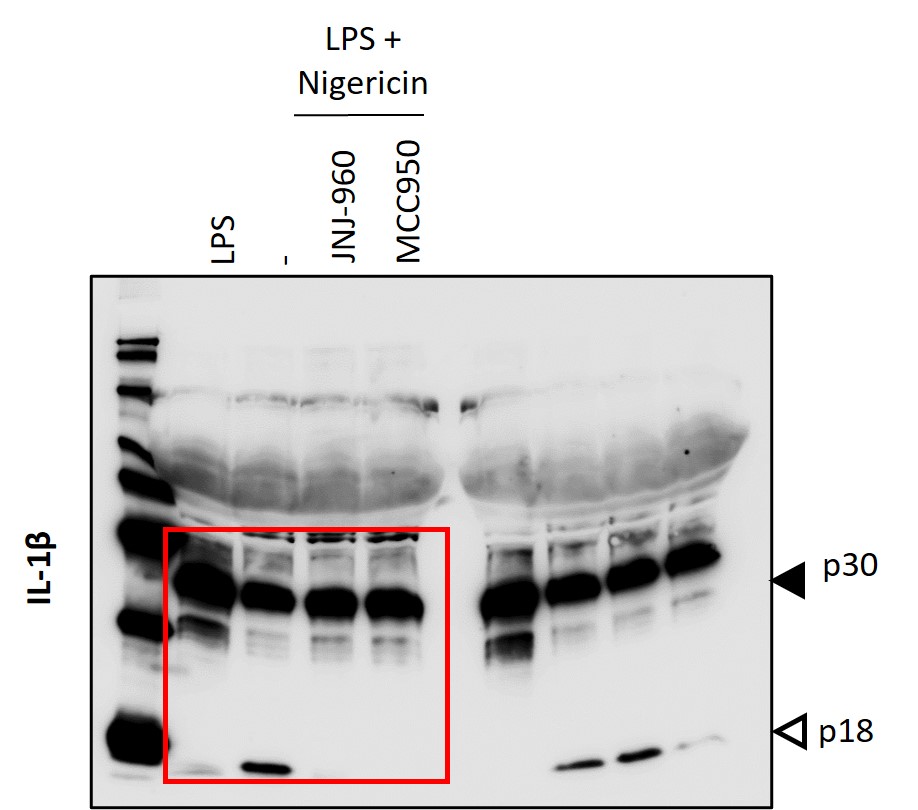

Supplement: Supplementary file 6 — Source data Fig. 4 [file 44321_2024_181_MOESM6_ESM.zip › Fig 4C_IL1b_Nig.jpg]

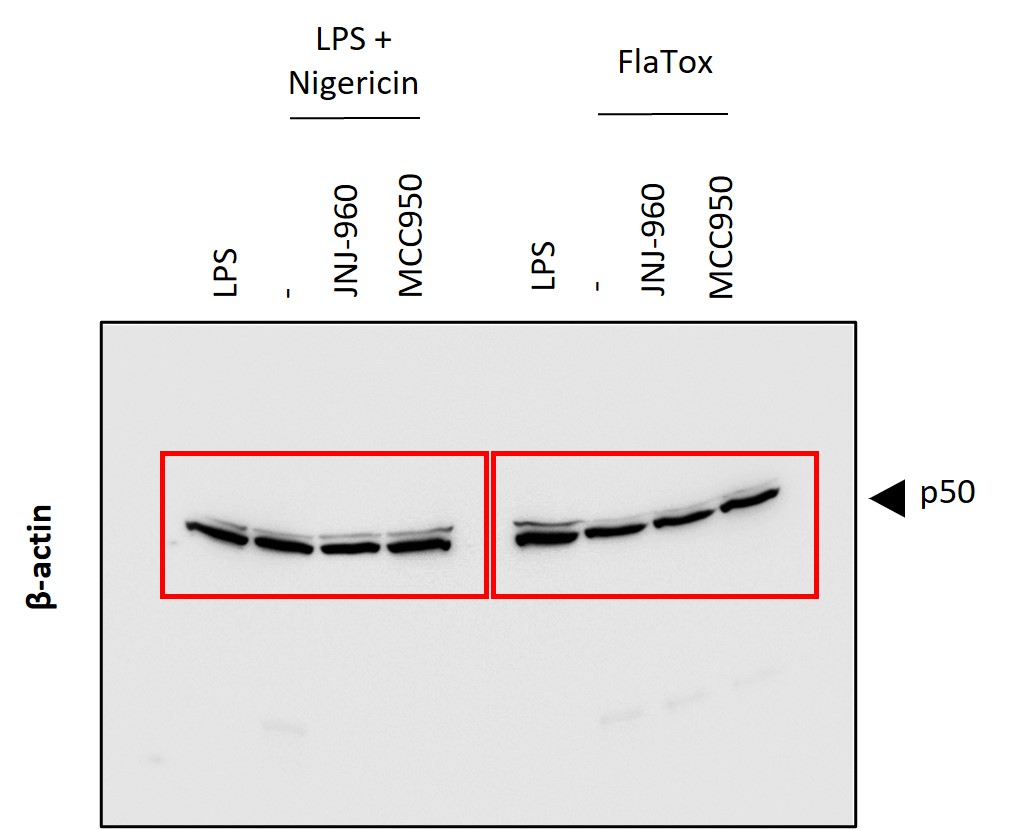

Supplement: Supplementary file 6 — Source data Fig. 4 [file 44321_2024_181_MOESM6_ESM.zip › Fig 4C-actin.jpg]

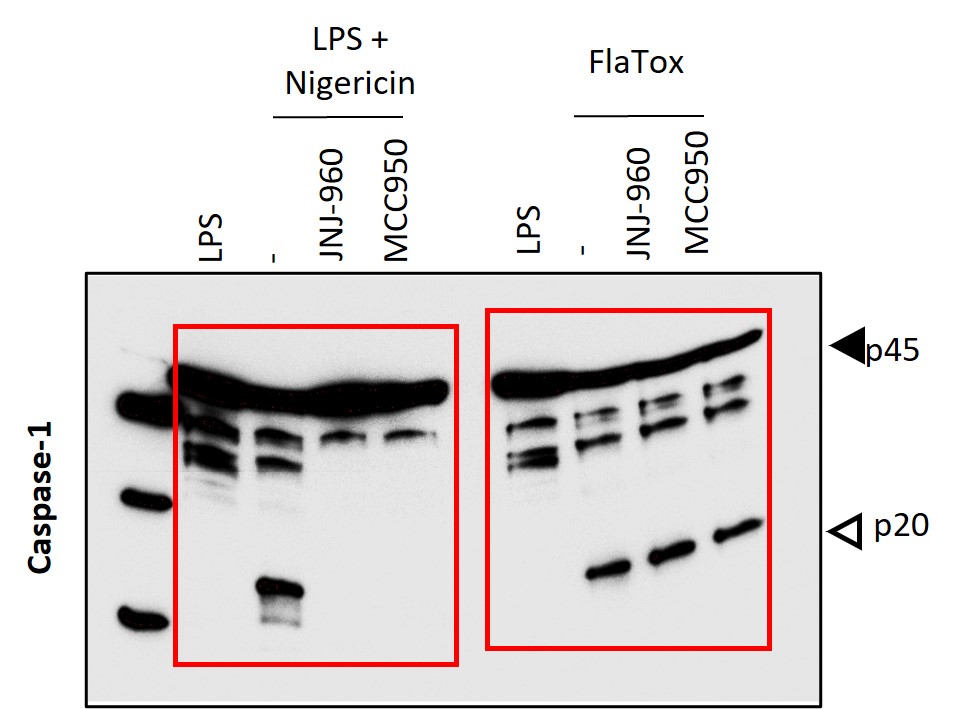

Supplement: Supplementary file 6 — Source data Fig. 4 [file 44321_2024_181_MOESM6_ESM.zip › Fig 4C-Casp1.jpg]

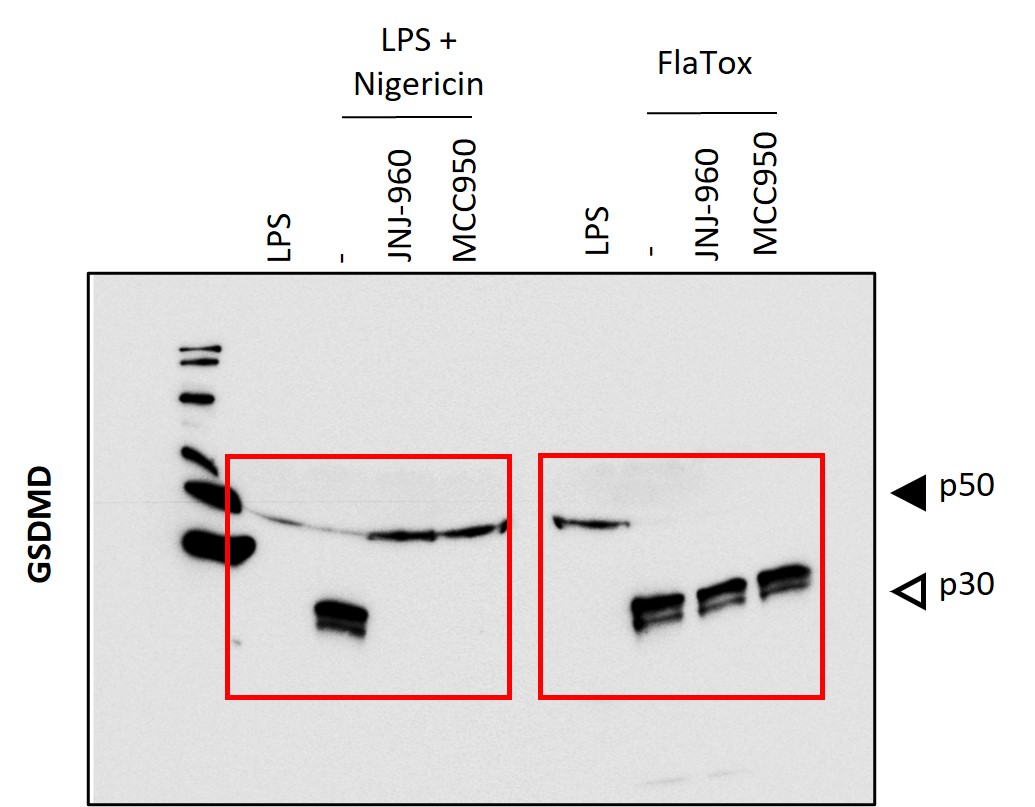

Supplement: Supplementary file 6 — Source data Fig. 4 [file 44321_2024_181_MOESM6_ESM.zip › Fig 4C-GSDMD.jpg]
