# Supplementary material for: Navigating from cellular phenotypic screen to clinical candidate: selective targeting of the NLRP3 inflammasome
Source: EMBO Mol Med. 2024 Dec 9;17(1):54–84. doi: 10.1038/s44321-024-00181-4 (PMC11730736; doi:10.1038/s44321-024-00181-4)
Supplement: Supplementary file 8 — Expanded View Figures [file 44321_2024_181_MOESM8_ESM.pdf]

Expanded View Figures

Figure EV1. Structural insights into NLRP3 small-molecule comparison and proposed Compound A binding mode.

(**A**) Schematic representation of NLRP3 architecture, with domains colored as follows: PYD (red), NBD (orange), HD1 (slate), WHD (light green), HD2 (dark green), and LRR (light gray). (**B**) NLRP3 small-molecule inhibitors were aligned using the central amine moiety as the reference point. The PDB IDs for each small-molecule-bound NLRP3 structure were shown. (**C**) Key interacting residues of NLRP3 with the small molecule are highlighted and color-coded as in Fig. EV1A. Note that the residues are grouped by subdomains but may not precisely represent the actual spatial interactions with NLRP3. Interacting residues of NLRP3 with compound A, observed in the simulation (Frame 50), are highlighted with dashed square. (**D**) Pairwise structural comparison of small-molecule binding to NLRP3. The small molecules are depicted as sticks in pink or yellow, while NLRP3 is shown as a cartoon, color-coded as in Fig. EV1A. Key interacting residue side chains are represented as lines, with colors corresponding to their respective subdomains. (**E**) The compound-accessible surface area was plotted against the NLRP3 area changes caused by compound binding (in $Å^2$). These areas and their differences were calculated using the AreaMol program from the CCP4 package. Values derived from real complex structures are shown in black spheres, while those from simulations (Frame 50, 500, and 1000) are represented in pink and yellow spheres for compounds C and A, respectively. (**F**) Violin plot showing distances between Val353 and Glu629, and between compounds A or C and Arg578 (a) or Glu636 (b) from the entire simulation set. Insets display the measured distances between compounds A or C and Arg578 (a) or Glu636 (b). Simulations for Apo, Compound A, and C are colored gray, yellow, and magenta, respectively. Slate and red bars indicate the distances of Val353 and Glu629 from NLRP3's closed (PDB IDs: 7PZC, 7VTP, 7VTQ, 8SWK, 8SXN, 8ETR, 7ALV, 8WSM, 9DH3, and 8RI2) and open structures (PDB IDs: 8SWF and 8EJ4). MD simulations were conducted three times ($n = 3$).

 © The Author(s)

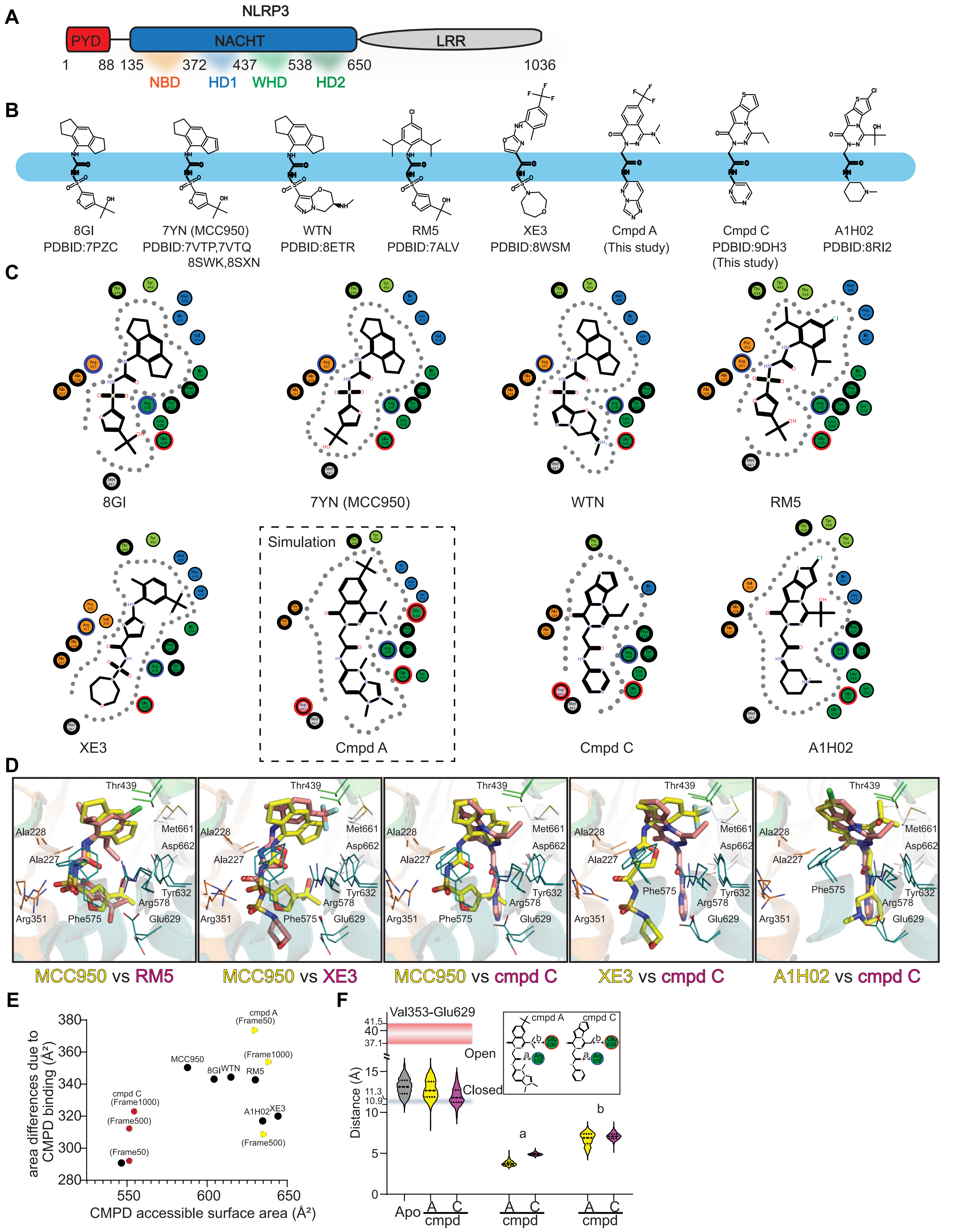
A
NLRP3
PYD
NACHT
LRR
1 88 135 372 437 538 650 1036
NBD HD1 WHD HD2
B
8GI PDBID:7PZC
7YN (MCC950) PDBID:7VTP,7VTQ 8SWK,8SXN
WTN PDBID:8ETR
RM5 PDBID:7ALV
XE3 PDBID:8WSM
Cmpd A (This study)
Cmpd C PDBID:9DH3 (This study)
A1H02 PDBID:8RI2
C
8GI
7YN (MCC950)
WTN
RM5
XE3
Simulation
Cmpd A
Cmpd C
A1H02
D
Thr439
Ala228
Ala227
Met661
Asp662
Tyr632
Arg578
Arg351
Phe575
Glu629
MCC950 vs RM5
MCC950 vs XE3
MCC950 vs cmpd C
XE3 vs cmpd C
A1H02 vs cmpd C
E
area differences due to CMPD binding (Å²)
CMPD accessible surface area (Å²)
cmpd A (Frame50)
(Frame1000)
MCC950
8GI
WTN
RM5
cmpd C (Frame1000)
(Frame500)
(Frame50)
A1H02
XE3
(Frame500)
380
360
340
320
300
280
550
600
650
F
Val353-Glu629
Open
Closed
Distance (Å)
41.5
40
37.1
15
11.3
10.9
10
5
0
cmpd A
cmpd C
Apo
A C cmpd
A C cmpd
A C cmpd
a
b

A

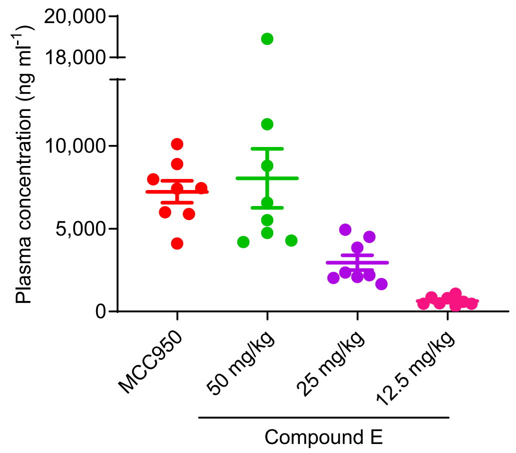

B

	[Total Plasma] nM	[Free plasma] nM
Cmpd E (50 mg/Kg)	22,022	264
Cmpd E (25 mg/Kg)	8079	96
Cmpd E (12.5 mg/Kg)	1745	21

C

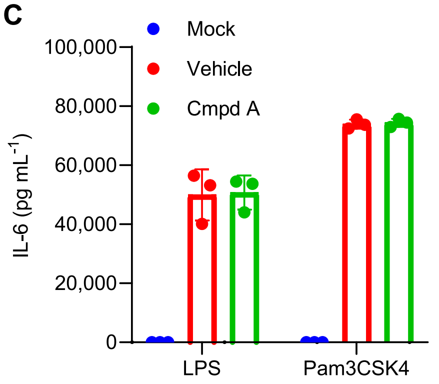

D

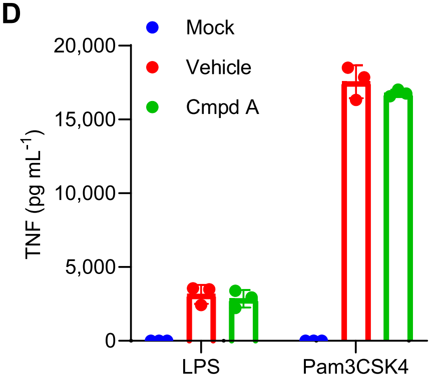

E

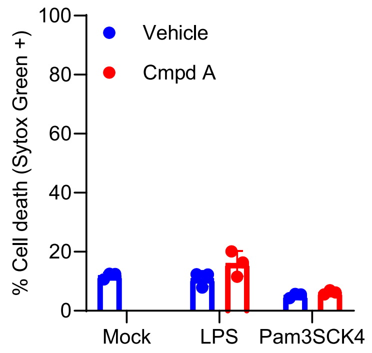

F

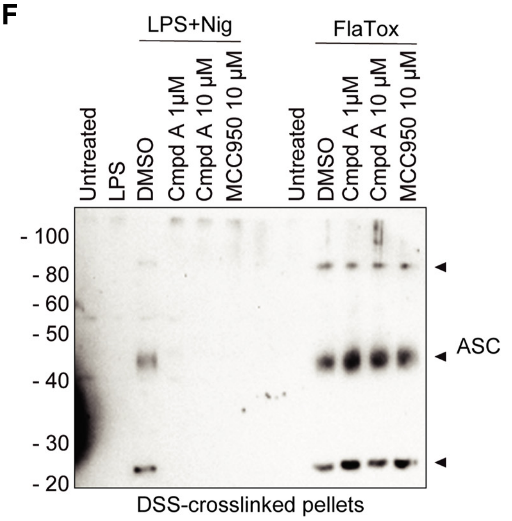

G

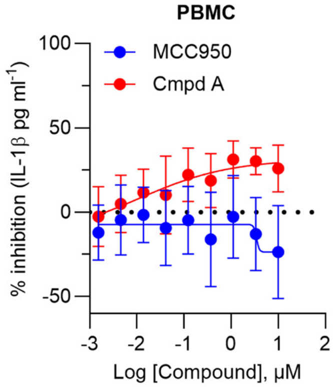

H

	Human %PPB	Murine %PPB
MCC950	3.81	1.54
Cmpd A	0.38	2.03

I

CD1 mice 5 mg/kg PO PEG400	Cmax,u (nM,u)	[plasma], u @4h (nM,u)	T1/2 (h)	T max (h)
Cmpd A	314	64	1.56 ± 0.38	0.5 ± 0.00

J

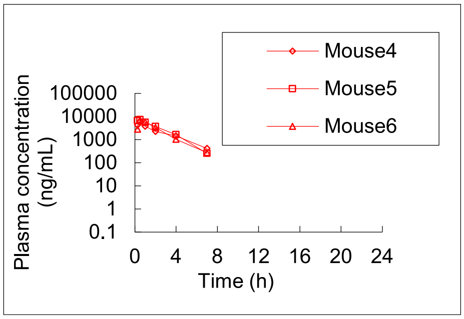

 © The Author(s)

Figure EV2. Characterization of compound E and A.

(**A**, **B**) C57BL/6 mice were pretreated with reference compound MCC950 or compound E for 30 min followed by LPS exposure for 4 h. Blood collected and compound concentration determined in plasma. Calculation of free compound levels based on plasma-protein binding. Per group 8 animals were used and mean +/- SEM is depicted. Free plasma levels are calculated based on protein binding. (**C**, **D**) LPS or Pam3CSK4-induced NFκB led to the production of IL-6 (**C**) and TNF (**D**) which was not impacted by pretreatment with compound A. Representative image from $n = 3$ shown with 3 technical repeats and mean +/− SD depicted. (**E**) Cell death induced by 16 h treatment with LPS or Pam3CSK4 in combination with compound A. Representative image from $n = 3$ shown with 3 technical repeats and mean +/− SD depicted. (**F**) Wild-type BMDMs either primed with LPS or left untreated for 2 h in presence of Cmpd A (1–10 μM) or MCC950 (10 μM) were stimulated with Nigericin (Nig) or FlaTox for 2 h. DSS-crosslinking of lysates was performed after stimulation and high-order oligomerisation of ASC was detected by immunoblotting. Western blot is representative image of two independent experiments ($n = 2$). (**G**) Pam3csk4-primed human PBMCs were treated with reference compound and compound A for 30 min followed by NdlTox stimulation for 3 h. Supernatant is collected and used for cytokine detection on MSD. PBMCs from 3 independent donors ($n = 3$) were used quadruplicate and all data pooled. Mean +/− SD is depicted. (**H**) % plasma-protein binding of reference compound and compound A. (**I**, **J**) Pharmacodynamic characteristics of compound A determined in CD1 mice.

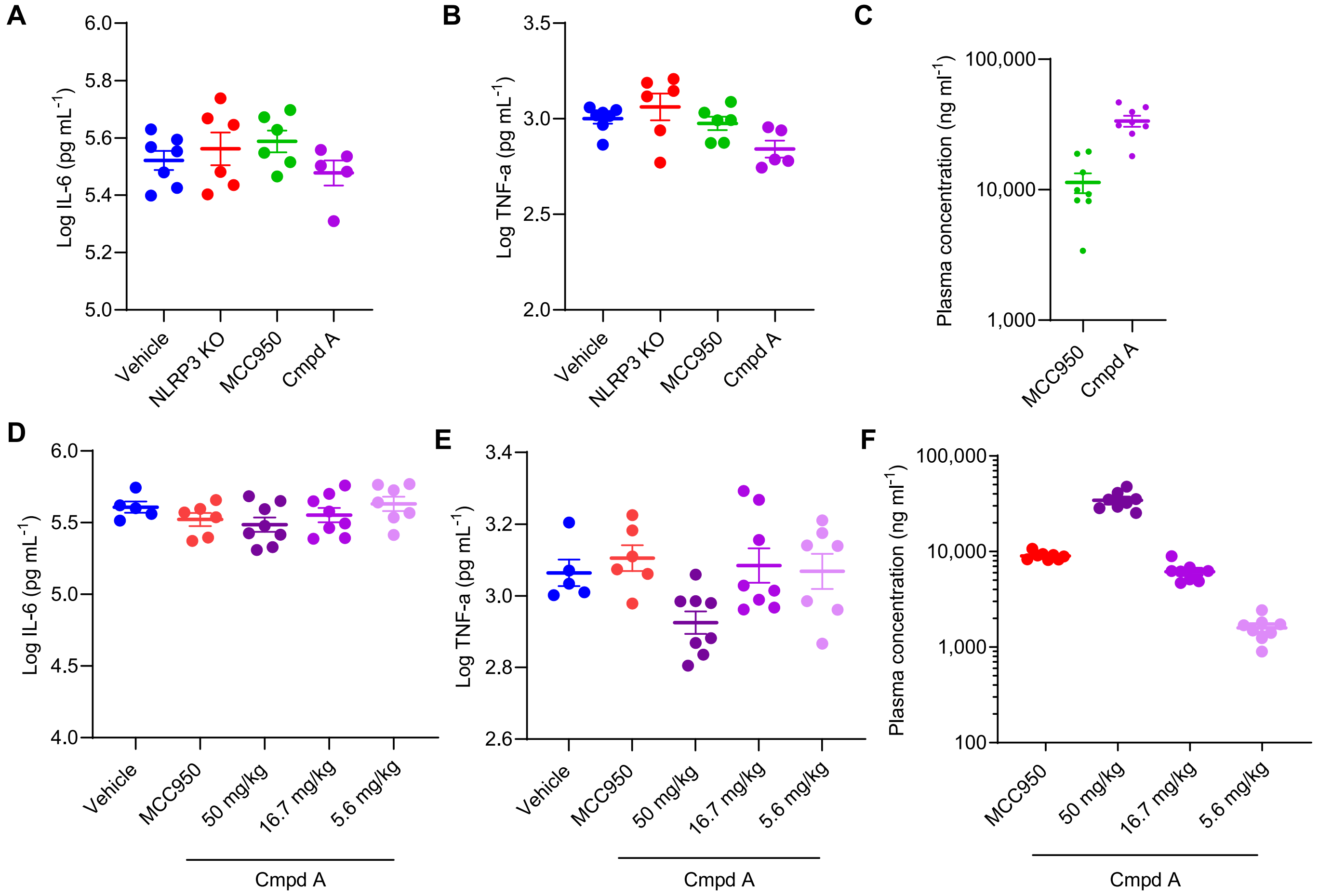

G

		ng/ml	nM	Free nM
Compound A	**50 mg/kg**	34337.50	79418.77139	1612.2
	**16.7 mg/kg**	6141.25	14204.0198	288.34
	**5.6 mg/kg**	1587.38	3671.41965	74.53

H

**Observation**	**Vehicle<br>0 mg/kg/day**	**Low<br>25 mg/kg/day**	**High<br>100 mg/kg/day**
**Feces with abnormal consistency**<br>- **Soft**<br>- **Liquid**	3/4 (D14): slight to moderate<br>3/4 (D14): slight	1/4 (D14): slight<br>1/4 (D14): slight	2/4 (D14): slight<br>2/4 (D14): slight
**Erected fur**	2/4 (D4-9)	3/4 (D3-D14)	4/4 (D5-D14)
**Generalized oily fur aspect**	-		1/4 (D3-D9)
**Color discharge, mouth**	-	1/4 (D10)	-
**Histopathology analysis of organs (heart, kidney, liver, spleen)**	4/4: normal heart<br>4/4: normal kidney<br>4/4: normal liver<br>4/4: normal spleen	4/4: normal heart<br>4/4: normal kidney<br>4/4: normal liver<br>4/4: normal spleen	4/4: normal heart<br>4/4: normal kidney<br>4/4: normal liver<br>4/4: normal spleen

Soft to liquid feces was observed in all groups including control on Day 14.
These signs are commonly seen in mice dosed orally with PEG400 and were thus considered vehicle-related.
-: no noteworthy observation

 © The Author(s)

Figure EV3. In vivo validation of compound A.

(**A**, **B**) Levels of IL-6 and TNF determined in C57BL/6 mice treated with compound followed by LPS injection for 4 h. Per group 8 animals were used and mean +/− SEM is depicted. (**C**) Compound levels determined in C57BL/6 mice treated with compound followed by LPS injection for 4 h. Per group 8 animals were used and mean +/− SEM is depicted. (**D**, **E**) Levels of IL-6 and TNF determined in C57BL/6 mice treated with different doses of compound followed by LPS injection for 4 h. Per group 8 animals were used and mean +/− SEM is depicted. (**F**, **G**) Compound levels determined in C57BL/6 mice treated with compound followed by LPS injection for 4 h. Calculation of free compound levels at endpoint. Per group 8 animals were used and mean +/− SEM is depicted. (**H**) Toxicology profile of compound A after a preclinical short 14 d tox study in mice. Per group, 4 animals are used.

© The Author(s)

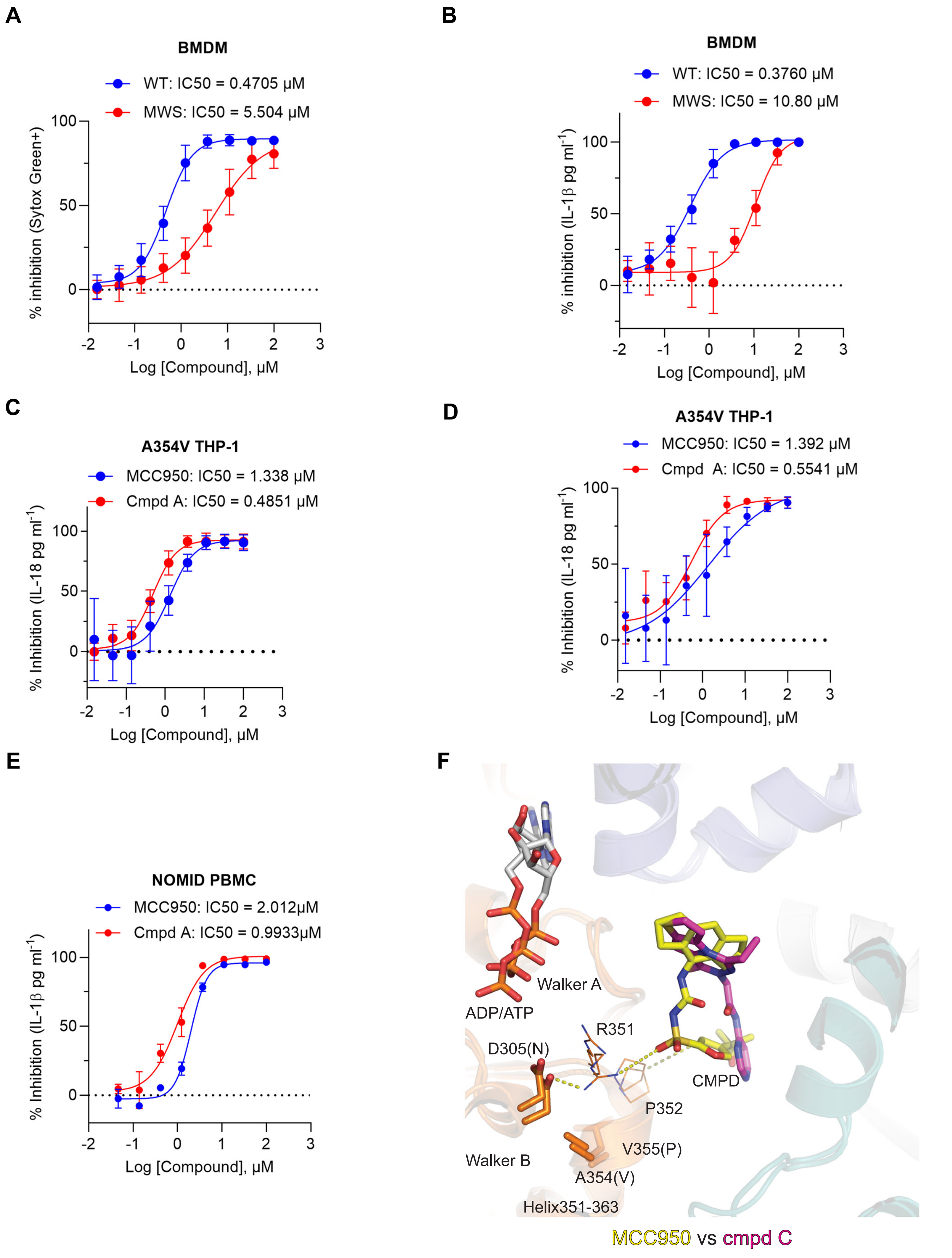
A
BMDM
WT: IC50 = 0.4705 µM
MWS: IC50 = 5.504 µM
% inhibition (Sytox Green+)
Log [Compound], µM
B
BMDM
WT: IC50 = 0.3760 µM
MWS: IC50 = 10.80 µM
% inhibition (IL-1β pg ml-1)
Log [Compound], µM
C
A354V THP-1
MCC950: IC50 = 1.338 µM
Cmpd A: IC50 = 0.4851 µM
% Inhibition (IL-18 pg ml-1)
Log [Compound], µM
D
A354V THP-1
MCC950: IC50 = 1.392 µM
Cmpd A: IC50 = 0.5541 µM
% Inhibition (IL-18 pg ml-1)
Log [Compound], µM
E
NOMID PBMC
MCC950: IC50 = 2.012µM
Cmpd A: IC50 = 0.9933µM
% Inhibition (IL-1β pg ml-1)
Log [Compound], µM
F
Walker A
ADP/ATP
R351
D305(N)
CMPD
P352
V355(P)
Walker B
A354(V)
Helix351-363
MCC950 vs cmpd C

 © The Author(s)

Figure EV4. Impact of CAPS mutations on the efficacy of compound A.

(**A**, **B**) Wild-type or conditional MWS BMDMs were treated with a dose response of MCC950 and effect on cell death (**A**) and IL-1β release (**B**) was determined. BMDMs from 3 independent animals ($n = 3$) were used in duplicate and pooled data depicted as mean +/− SD. (**C**, **D**) Undifferentiated THP-1 cells were treated with doxycycline to allow expression of A354V mutant NLRP3 whereafter cells were treated with reference compound or compound A in dose response followed by treatment with LPS (**A**) or LPS + Nig (**B**). Supernatant was collected and IL-18 detected using Luminex. Two independent repeats ($n = 2$) were performed in quadruplicate and pooled data depicted as mean +/− SD. (**E**) PBMCs from a patient with NOMID mutation were treated with reference compound or compound A followed by LPS + Nig. Supernatant was collected and IL-1β measured by MSD. PBMCs were treated in duplicate, and data represented as mean +/− SD. (**F**) Comparison of MCC950 and compound C binding to human NLRP3. Small molecules are shown as pink (compound C) and yellow (MCC950) sticks, while NLRP3 is depicted as a color-coded cartoon, as in Fig. EV1A. Side chains of Arg351 and Pro352 are displayed as lines, and mutations from CAPS (Ala354Val), FCAS (Leu355Pro), and NOMID (Asp305Asn) patients are represented as sticks, colored according to their respective subdomains. Yellow dashed lines indicate potential interactions between residues and MCC950.

© The Author(s)

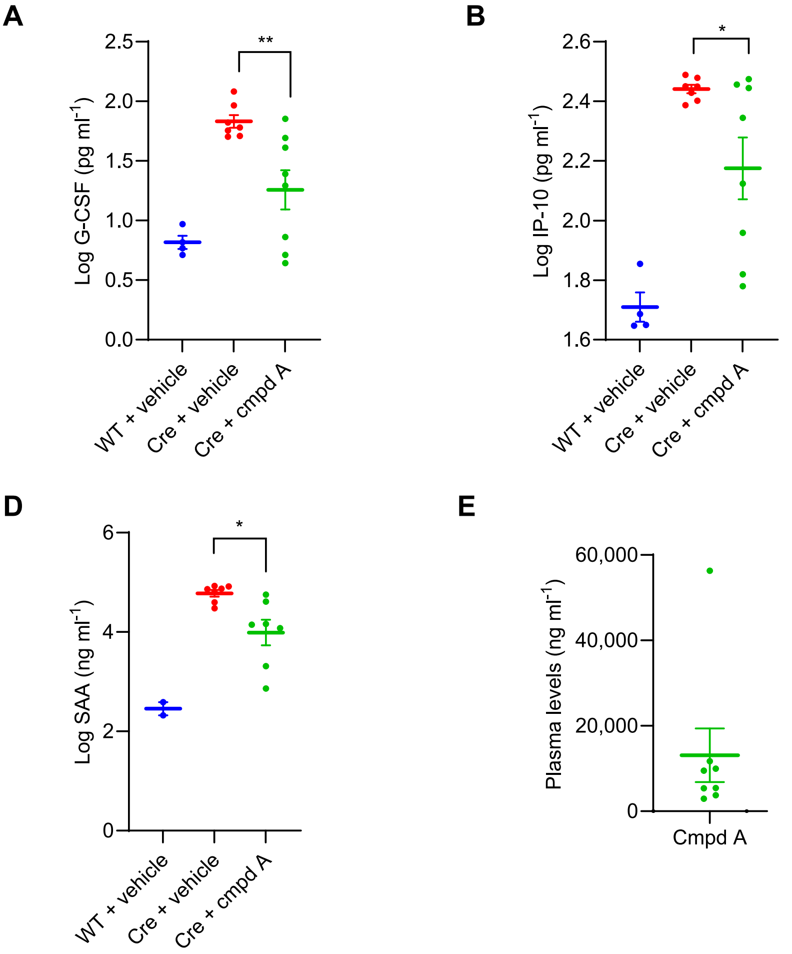

F

Exposure	Compound A
	100 mg/kg
ng/ml	13119
nM	30342.19
Free nM	615.95

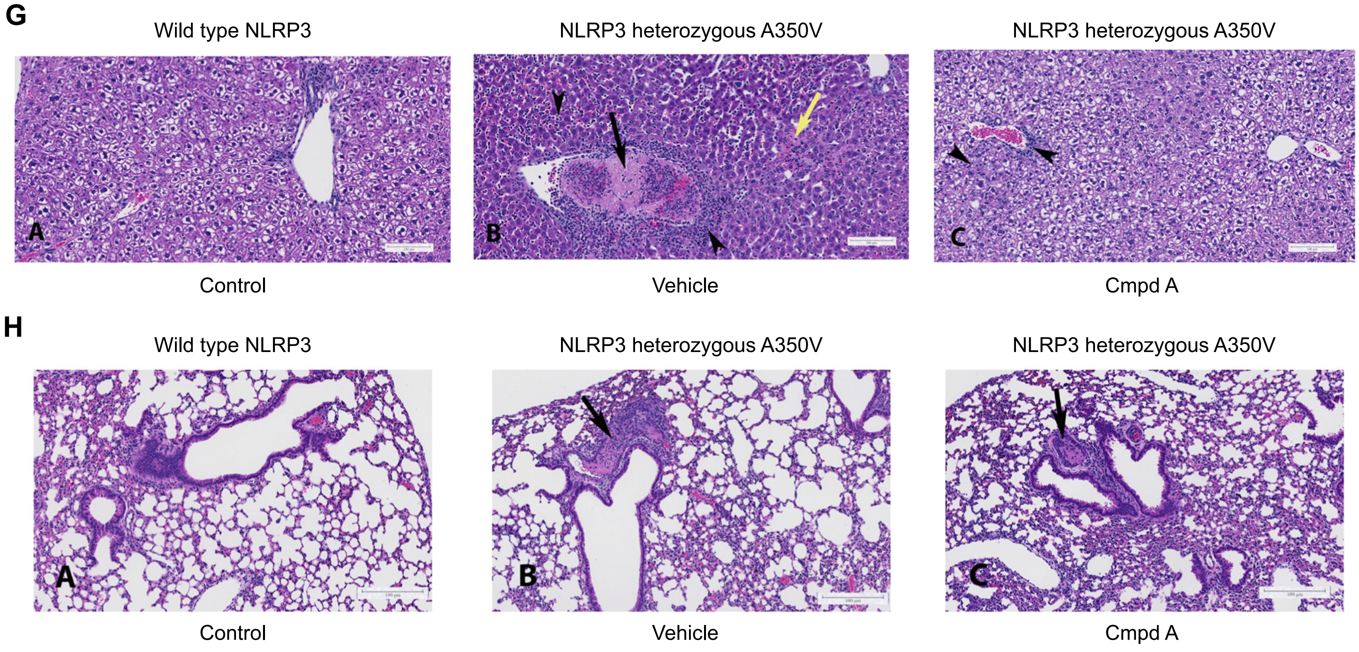

 © The Author(s)

Figure EV5. In vivo validation of compound A using the MWS mouse model.

(**A–D**) Tamoxifen-induced MWS-mediated disease was measured using G-CSF (**A**), IP-10 (**B**), IL-6 (**C**) and SAA (**D**) in plasma after collection of the blood on day 10. Per group 4 (WT vehicle), 7 (Cre + vehicle) or 8 (Cre + cmpd A) animals were used and mean +/− SEM is depicted. Unpaired two-tailed t-test with welch's correction in Cre treated vs vehicle samples was performed: $^{**}P = 0.0058$ (G-CSF), $^{*}P = 0.0317$ (IP-10), $^{*}P = 0.0145$ (IL-6), $^{*}P = 0.0145$ (SAA). (**E**, **F**) Levels of compound in plasma in blood collected at day 10, 24 h after last oral dosing. Eight animals were used in this group and mean +/− SEM is depicted. Free compound was calculated (**F**). (**G**) Photomicrograph (H&E) of liver sections from a control wildtype (A), vehicle-treated NLRP3 heterozygous (B) and compound A-treated NLRP3 heterozygous (c) mouse. Note the hepatocellular necrosis (yellow arrow), portal vein thrombosis (black arrow), and inflammatory cells infiltration in the sinusoids and portal triads (arrowheads) of the vehicle-treated liver. In contrast, compound A-treated liver appeared histologically comparable to wild-type liver and exhibited only minimal inflammatory cells infiltration (arrowheads) and significant reduction in the severity of thrombosis and necrosis. Bar= 100 microns. (**H**) Photomicrograph (H&E) of lung sections from a control wildtype (A), vehicle-treated NLRP3 heterozygous (B) and compound A-treated NLRP3 heterozygous (c) mouse. Compound A-treated lung appeared histologically comparable to wild-type liver and exhibited reduction in the severity of thrombosis (arrows) when compared to vehicle-treated NLRP3 heterozygous. Bar = 100 microns.

© The Author(s)
